# Evaluating Cognition Across Aging and Traumatic Brain Injury: Integrating Neurological and Neuropsychological Approaches

**DOI:** 10.3390/jcm15103822

**Published:** 2026-05-15

**Authors:** Miguel A. Pappolla, Sean L. Pappolla, Remi Nader, Mohammad K. Hamza, Felix Fang, Xiang Fang

**Affiliations:** 1 Department of Neurology, University of Texas Medical Branch, Galveston, TX 77555, USA; sxfang@utmb.edu; 2 University of Texas Medical Branch, Galveston, TX 77555, USA; sepappol@utmb.edu; 3 Department of Neurosurgery, University of Texas Medical Branch, Galveston, TX 77555, USA; 4 Counseling Department, Lamar University, Beaumont, TX 77710, USA; 5 Department of Physical Medicine and Rehabilitation, University of North Texas Health Science Center (UNTHSC), Texas Rehabilitation Hospital, Fort Worth, TX 76104, USA; felix.fang@texasrehabhospital.com

**Keywords:** aging, behavioral neurology, clinical neuropsychology, cognition, cognitive assessment, medicolegal evaluation, neurocognitive disorders, neurological examination, neuropsychological assessment, traumatic brain injury

## Abstract

**Background/Objectives:** The evaluation of cognition is central to many neurological conditions, including traumatic brain injury, Alzheimer’s disease, Lewy body disease, frontotemporal degeneration, and vascular disorders. In clinical practice, particularly in aging populations, cognitive complaints often arise in the context of mixed neurological processes, requiring careful integration of cognitive and non-cognitive findings. Despite this, there remains limited clarity regarding the respective roles of neurologists and clinical neuropsychologists and the distinction between cognitive and neuropsychological assessments, terms that are often used interchangeably despite important differences in methodology and scope. This lack of a shared framework has practical consequences. Cognitive test results, when interpreted in isolation for diagnosis, may be misconstrued as comprehensive measures of brain function, particularly when non-cognitive neurological features such as motor, cerebellar, or vestibular abnormalities should have been considered (but were not). **Methods:** In this narrative review, we synthesize clinical guidelines, consensus statements, regulatory sources, and representative empirical literature to articulate a competence-based framework in which cognitive assessment is a medically integrated process incorporating history, functional evaluation, neurological examination, and the targeted use of standardized neuropsychological instruments. **Results:** Neurologists are trained to establish medical diagnoses and integrate cognitive findings into the context of neurological disease, while neuropsychologists contribute detailed psychometric characterization, culturally and demographically informed interpretation, cognitive phenotyping, functional characterization, and validity assessment in complex clinical and medicolegal contexts. Although neuropsychologists are qualified to diagnose neurocognitive disorders using standardized diagnostic criteria, attribution to specific neurological etiologies requires a comprehensive medical evaluation that extends beyond cognitive testing alone. **Conclusions:** We outline a tiered approach to evaluation that aligns assessment methods with clinical questions and supports accurate diagnosis, interdisciplinary collaboration, and patient-centered care.

## 1. Introduction

Cognitive symptoms are among the most common reasons patients seek neurological evaluation, particularly in midlife and older age. Memory complaints, attention deficits, executive dysfunction, and slowed processing speed frequently accompany a wide range of neurological conditions, including traumatic brain injury, neurodegenerative disease, cerebrovascular pathology, systemic illness, medication effects, sleep disorders, and psychiatric comorbidity. As populations age, clinicians increasingly encounter patients in whom cognitive symptoms arise from overlapping or interacting etiologies rather than from a single, isolated disease process.

Traumatic brain injury (TBI) occupies a distinctive position within this landscape. Although often discussed separately from aging-related disorders, TBI frequently precedes or coexists with later-life cognitive decline and may interact with neurodegenerative, vascular, and neuropsychiatric processes over time. In older adults, a history of TBI may lower cognitive reserve, amplifying vulnerability to subsequent insults, and contribute to persistent non-cognitive neurological deficits that substantially affect daily functioning. These mixed presentations demand a diagnostic approach that integrates cognition within a broader neurological evaluation.

Despite the centrality of cognitive evaluation to neurological practice, confusion persists, particularly in interdisciplinary and medicolegal contexts, regarding who is qualified to assess cognition and how cognitive data should be interpreted. In some medicolegal settings, questions arise regarding the extent to which neurologists may evaluate cognition or interpret selected cognitive test data without psychology-based neuropsychology credentials. When framed exclusively as a credential-based dispute, this issue can shift attention away from the clinical question, the clinician’s demonstrated competence, and the need for diagnostic integration. The goal of this review is therefore not to privilege one discipline over another, but to clarify how different forms of expertise contribute to cognitively focused neurological diagnosis.

At the same time, it would be inaccurate to suggest that neurologists, as a group, receive the same depth of training as clinical neuropsychologists in psychometrics, test development, performance validity methodology, and normative interpretation. Neuropsychological assessment represents a specialized methodological skill with distinct and important strengths. A mature framework, therefore, should reject false dichotomies: cognitive assessment is a core neurologic competency; neuropsychological assessment is a specialized psychometric methodology; and collaboration, guided by competence and clinical context, best serves patients’ interests. Meaningful collaboration, however, requires more than parallel documentation; it depends on direct communication between disciplines to align concepts, clarify interpretive assumptions, and reduce the risk of misinterpretation.

This paper aims to clarify terminology, delineate roles, describe complementary contributions, and propose a practical evaluation approach that is clinically appropriate, scientifically grounded, and resistant to misinterpretation.

Review methodology, scope, and organization

This article is a narrative and conceptual review, not a systematic review or meta-analysis. To improve transparency, the review used a structured narrative approach. Literature was identified through targeted searches of PubMed/MEDLINE and Google Scholar, supplemented by review of professional, regulatory, and reimbursement sources (File S1), including guidance from the American Academy of Neurology, American Psychological Association, National Academy of Neuropsychology, American Academy of Clinical Neuropsychology, Accreditation Council for Graduate Medical Education, American Board of Psychiatry and Neurology, Centers for Medicare and Medicaid Services, AMA CPT coding framework, VA/DoD clinical practice guidelines, DSM-5, and dementia consensus statements. Search terms included combinations of cognitive assessment, cognitive testing, neuropsychological assessment, neurobehavioral status examination, dementia diagnosis, mild cognitive impairment, traumatic brain injury, mild traumatic brain injury, performance validity, biomarker, neurological examination, cross-cultural neuropsychology, education, language, socioeconomic factors, medicolegal evaluation, capacity, fitness for duty, digital cognitive assessment, and artificial intelligence. Sources were selected when they were directly relevant to terminology, scope of practice, professional competence, diagnostic integration, dementia, aging, TBI, or interpretation of cognitive and neuropsychological test findings. Priority was given to English-language clinical guidelines, consensus recommendations, position papers, systematic reviews, meta-analyses, professional practice standards, regulatory documents, and representative empirical studies. Because the central question concerns terminology, professional competence, and diagnostic integration rather than the pooled diagnostic accuracy of a single cognitive instrument, a quantitative meta-analysis was not performed and a PRISMA-style flow diagram was not generated. Searches did not identify a large-scale meta-analysis directly comparing the diagnostic accuracy of neurologists and neuropsychologists as professional groups; the available diagnostic-accuracy literature generally evaluates instruments, batteries, biomarkers, or multidisciplinary memory-clinic models rather than professional title as an isolated predictor of diagnostic accuracy. Where direct comparative evidence was unavailable, statements about professional roles are presented as an interpretive synthesis of guidelines, training standards, regulatory frameworks, and clinical reasoning rather than as pooled empirical effect estimates.

The review is organized sequentially. First, it defines cognitive testing, cognitive assessment, and neuropsychological assessment and distinguishes screening from comprehensive neuropsychological evaluation. Second, it addresses competence, training pathways, and the complementary contributions of neurologists and clinical neuropsychologists. Third, it explains why neurological examination, biomarkers, and non-cognitive neurological findings are essential for etiologic attribution, using dementia, aging, and traumatic brain injury as model contexts. Finally, it synthesizes these points into a practical tiered model, clarifies the scope of medicolegal implications, and summarizes limitations.

**Definitions and Conceptual Approach to Diagnosis**

**Terminology and sources of confusion**

In everyday clinical language, the terms cognitive testing, cognitive assessment, and neuropsychological assessment are often used interchangeably. Cognitive testing may range from a brief screening instrument, such as the Mini-Mental State Examination, to an extended battery of standardized measures, while neuropsychological assessment is sometimes used broadly to refer to any formal use of a set of cognitive instruments [1]. Although such usage is common, it obscures important conceptual distinctions.

The ambiguity becomes consequential when cognitive findings are interpreted outside their intended scope. This concern is not unique to the neurology-neuropsychology interface. Professionals across disciplines, including speech-language pathologists, occupational therapists in rehabilitation settings, and clinical psychologists without specialized neuropsychological training, increasingly conduct cognitive testing in clinical practice. When such testing is performed without adequate understanding of psychometric limitations or neuropathological correlates, we often see misattribution of cognitive findings to psychiatric or developmental conditions while overlooking neurological etiologies.

Another problem arises from methodological differences that may be mistaken for differences in professional authority, and from training differences that may be misrepresented as differences in diagnostic legitimacy. A clear conceptual framework is therefore necessary to distinguish what is being done from who is doing it.

Rather than treating cognitive assessment and neuropsychological assessment as interchangeable labels, it is more accurate to understand them as related but distinct constructs situated along a continuum of cognitive evaluation [1].

**Cognitive assessment as a clinically integrated concept**

Cognitive assessment refers to a structured medical evaluation of cognitive functioning that is integrated with neurological and systemic context. Its defining feature is not the use of any single instrument or battery, but the synthesis of cognitive observations with clinical history, functional status, neurological examination, and relevant medical data to answer diagnostic and management questions [2].

In aging populations, these questions commonly include whether cognitive change reflects normal aging, mild cognitive impairment, neurodegenerative disease, vascular cognitive impairment, effects of systemic illness or medication, sequelae of traumatic brain injury, or mixed etiologies. Accordingly, a cognitive assessment is inherently contextual rather than purely psychometric.

Medication effects, sleep disorders, sleep deprivation, untreated obstructive sleep apnea, pain, fatigue, sensory loss, psychiatric symptoms, socioeconomic disadvantage, literacy, educational quality, and linguistic or cultural factors should be considered as potential modifiers of test performance. These factors do not invalidate cognitive assessment; rather, they determine the level of assessment needed and may favor referral for comprehensive neuropsychological evaluation when standard administration, normative interpretation, or validity judgments are complicated. Their detailed treatment as independent causes of cognitive impairment is beyond the scope of this review, but their recognition is part of the integrated neurological framework proposed here [2].

**Cross-cultural, educational, and socioeconomic considerations**

Culture, language, literacy, quality of education, acculturation, interpreter use, and socioeconomic context can influence cognitive-test performance and the appropriateness of available norms. These considerations apply to both neurologist-led cognitive assessment and comprehensive neuropsychological assessment. In complex cases, especially when the patient is bilingual, has limited formal education, is tested outside the language or culture in which normative data were developed, or has sensory or motor limitations, specialized neuropsychological methods and culturally appropriate normative interpretation may be required to reduce the risk of misclassification [3,4].

The American Academy of Neurology (AAN) Behavioral Neurology Section Workgroup developed the concept of the Neurobehavioral Status Exam (NBSE), which involves domain-specific cognitive tests (such as those targeting memory or language) that together provide a more thorough diagnostic evaluation than general screening instruments alone [5]. This workgroup surveyed 200 behavioral neurologists about their clinical cognitive testing practices and identified 5–15 tests per cognitive domain suitable for office-based neurological practice, emphasizing tests with normative data and validation in neurologic disorders [5]. However, behavioral neurologists represent a small subset of clinicians who evaluate cognitive complaints. In practice, cognitive evaluations are generated across diverse disciplines, including geriatric medicine, rehabilitation medicine, psychiatry, and general neurology, reinforcing the importance of competence-based standards rather than title-based restrictions [1,5].

The AAN MCI Practice Guideline recommends that when a brief cognitive assessment is positive, patients should undergo more detailed cognitive evaluation, such as neuropsychological testing interpreted in the context of appropriate normative data, to formally establish the diagnosis, but notably does not restrict who performs this testing [6].

In practice, cognitive assessment typically incorporates history, collateral information, neurological examination and behavioral observation, targeted cognitive instruments, and integration with medical data such as imaging, electrophysiology, vestibular testing, sleep studies, and laboratory evaluations [2]. Importantly, cognitive assessment is not inherently brief; depending on the clinical question, it may be extensive and may incorporate standardized neuropsychological instruments when appropriate.

**Neuropsychological assessment as a psychometric methodology**

Neuropsychological assessment is a specialized form of cognitive evaluation distinguished primarily by its measurement model. It consists of a comprehensive, standardized assessment of cognitive and related behavioral functions, using instruments with norm-referenced data, typically spanning multiple domains.

The National Academy of Neuropsychology has described comprehensive neuropsychological assessments as inherently multidimensional, serving purposes that include identifying primary and secondary diagnoses, characterizing the nature and severity of cognitive difficulties, determining functional limitations, and guiding treatment and rehabilitation planning [7]. Core features include standardized administration and scoring, norm-referenced interpretation, attention to reliability and validity constraints, performance validity assessment when interpretability is a concern, fine-grained cognitive profiling, and translation of findings into functional and rehabilitative recommendations.

According to the APA Guidelines for Psychological Practice with Older Adults, a neuropsychological evaluation involves the objective measurement of cognitive performance through standardized psychometric instruments across multiple cognitive domains, with results integrated alongside information from collateral sources such as family members, friends, or caregivers, as well as historical, neurological, psychiatric, and other medical data [8]. Neuropsychologists with these competencies compare test performance against culturally and demographically appropriate normative data to determine whether the cognitive profile reflects expected patterns of later-life functioning or represents a meaningful decline from the individual’s baseline abilities [8].

Neuropsychological assessment can support DSM-based diagnosis of neurocognitive disorders, differential cognitive phenotyping, functional characterization, and clinically meaningful diagnostic hypotheses. However, test performance alone does not establish a specific medical or neurological etiology. Etiologic attribution requires integration with neurological, medical, functional, and contextual data [2].

**Evidence-based distinctions between cognitive screening and comprehensive neuropsychological assessment**

The evidence base distinguishes cognitive screening and comprehensive neuropsychological assessment along several fundamental dimensions as follows (Table 1):

This table is intended to clarify method and clinical purpose, not to assign exclusive ownership of cognitive evaluation to a single profession.

A. **Purpose and Scope:** Cognitive screening is designed to identify global impairment with adequate sensitivity but relatively low diagnostic specificity, serving primarily as a detection or screening tool [7]. The National Academy of Neuropsychology has explicitly stated that screening tests can be helpful for identifying individuals who need more comprehensive assessment and for monitoring treatment outcomes, but has emphasized that they should not be used as a substitute for comprehensive neuropsychological testing [7].
B. **Methodological Complexity:** Brief mental status examinations, such as the MMSE or the Montreal Cognitive Assessment, are short and narrow in scope and can be administered during routine clinical visits. The NBSE, developed by the AAN Behavioral Neurology Section, represents a more comprehensive level than a brief screening designed for office-based neurological practice [5]. It is sufficient in many cases, but it may fall short in circumstances that require more advanced psychometric methods, as outlined below (e.g., illiteracy or sensory or neurological deficits that prevent the performance of certain evaluative tasks in the NBSE or other batteries).
C. **Interpretive Framework:** Brief mental status tests demonstrate adequate sensitivity and specificity for dementia but perform poorly for mild cognitive impairment [9]. Importantly, these tools lack sensitivity to the subtle cognitive changes characteristic of pre-clinical stages, and there may be limited correspondence between a brief mental status score and the patient’s actual functional status [9]. Screening instruments are further limited by ceiling and floor effects; individuals with high premorbid ability may perform normally despite meaningful decline, while those with low baseline functioning, such as longstanding learning disabilities, may fail items for reasons unrelated to neurodegenerative illness. Neuropsychological assessment employs profile analysis to differentiate possible sources of cognitive impairment, requiring integration of standardized test performance with culturally, premorbid, and demographically appropriate normative data.

The APA Guidelines for Psychological Practice with Older Adults note that distinguishing among the various factors contributing to cognitive impairment in older adults is considerably more challenging and frequently necessitates a comprehensive neuropsychological evaluation [8]. Furthermore, both positive and negative results on brief mental status testing may warrant follow-up with a more detailed neuropsychological assessment, depending in the particular case [6,7].

**Competence Rather Than Professional Title**

Whether a clinician is qualified to perform or interpret a neuropsychological assessment should not be determined solely by professional title. The appropriate standard is competence with respect to the specific procedures performed. Competence is procedure-specific and context-dependent. It requires appropriate neuroscience education and training and experience with the instruments used, adherence to standardized administration and scoring procedures, understanding of psychometric limitations, and appropriate integration of findings with clinical context. This standard applies across professions.

The competence-based formulation used here is an interpretive synthesis of professional guidelines, training standards, and regulatory/reimbursement structures. It should not be read as a claim that any profession has blanket competence across all cognitive or neuropsychological methods. Nor is it intended to imply hierarchy, exclusivity, or interchangeability among disciplines. Competence remains method-specific, patient-specific, and question-specific, and must be judged in relation to the clinical question, the instruments used, the patient population, and the setting in which the evaluation occurs.

The APA guidelines emphasize that psychologists must recognize the distinctions among cognitive screening, cognitive testing, and neuropsychological testing and must critically evaluate whether their own training and education provide the requisite competency for each type of evaluation before performing it [1,8].

A physician is not automatically competent to administer or interpret complex psychometric batteries without relevant training, just as a neuropsychological battery alone (even if administered by a neuropsychologist) is insufficient to determine medical etiology or diagnosis without specialty-level neurological evaluation. Titles do not substitute for training or skills, and method does not substitute for diagnosis.

**Training and Competency Pathways**

**Neurology training and cognition**

Neurology is the medical specialty dedicated to disorders of the nervous system, and cognitive evaluation is intrinsic to its practice. Neurology training includes required exposure to behavioral neurology and the neurology of aging, as well as structured experience in psychiatry with explicit attention to cognition and behavior [10]. These requirements reflect the expectation that neurologists will routinely evaluate cognitive complaints and distinguish neurological disease from psychiatric, systemic, and functional contributors.

Neurology training emphasizes integration rather than isolated test interpretation. The diagnostic goal is to link the cognitive findings with the neurological examination, functional history, imaging, and ancillary testing, a skill set particularly relevant in aging populations and TBI, where cognitive symptoms are often influenced by non-cognitive neurological dysfunction. The importance of integrating cognitive findings with neurological and systemic data can be illustrated through representative clinical examples encountered in aging populations and in patients with traumatic brain injury.

In clinical practice, an older adult may present with progressive memory complaints and executive dysfunction. Cognitive screening suggests mild impairment, and subsequent neuropsychological testing identifies deficits in attention and executive domains. Based on cognitive data alone, the findings could be interpreted as consistent with early Alzheimer’s disease or a dysexecutive neurodegenerative process. However, further evaluation reveals critical features not captured by testing. A caregiver interview discloses recurrent visual hallucinations and marked fluctuations in cognition over the course of the day, features not reported by the patient. Neurological examination demonstrates asymmetric rigidity and bradykinesia. When these findings are integrated, the clinical picture is more consistent with dementia with Lewy bodies.

The reliance on caregiver history in this context is essential. Patients with fluctuating cognition or impaired insight may underreport or fail to recognize hallucinations and variability in function. Collateral information provides a more reliable account of symptom evolution over time and enables the detection of features that are diagnostically central but not accessible through patient self-report or structured cognitive testing. Without incorporating caregiver observations, the diagnosis in this case would likely be misattributed, with direct implications for prognosis and management.

In a second example, a middle-aged patient with a history of traumatic brain injury reports persistent cognitive complaints, including slowed processing and impaired concentration. Neuropsychological testing reveals mild reductions in processing speed and attention, findings that may be interpreted as nonspecific or within expected variability depending on premorbid functioning. However, the neurological examination reveals impaired smooth pursuits and conversion insufficiency, consistent with central oculomotor dysfunction. Functional history reveals difficulty with visually mediated tasks and increased symptom burden in complex sensory environments. When these findings are integrated, the patient’s cognitive complaints are better understood as emerging from TBI-related dysfunction rather than from unrelated cognitive decline.

These examples highlight a central pitfall of relying on cognitive or neuropsychological data in isolation. Cognitive test results represent measurements of performance under specific conditions and do not directly establish etiology. One common pitfall occurs when cognitive findings are interpreted without consideration of neurological or functional data. For example, a patient with traumatic brain injury may perform within normal limits on standardized testing yet exhibit impaired eye movements and reduced tolerance for cognitively demanding environments. If interpretation is based solely on test scores, the clinician may conclude that no neurological impairment is present, leading to dismissal of symptoms or misattribution to non-neurological causes. In reality, dysfunction resides in neural systems that are not adequately captured by the testing paradigm. Accordingly, cognitive assessment should be understood as one component of a broader diagnostic process.

The JAMA review on dementia diagnosis emphasizes that the evaluation of possible dementia requires a focused medical history together with a cognitive and neurologic examination, with the clinical history remaining the single most important diagnostic tool [11]. The neurologic examination serves to identify objective evidence of neurocognitive dysfunction, including aphasia, apraxia, and agnosia, and may reveal focal neurologic signs or parkinsonism, as is typically seen in the early stages of Lewy body disease [11].

**Board certification and cognition**

Neurology board certification further underscores the centrality of cognition within neurological practice. Certification content explicitly includes neuropsychological and cognitive testing among diagnostic procedures relevant to neurology, listed alongside other modalities such as vestibular testing, electrophysiology, autonomic studies, sleep studies, and neuroimaging. This structure reflects the expectation that neurologists understand and appropriately apply cognitive and neuropsychological testing as part of diagnostic reasoning [12].

**Subspecialization within neurology**

Beyond general training, many neurologists pursue subspecialization in behavioral neurology, cognitive neurology, dementia, and related fields. The Joint Committee on Subspecialty Certification of the American Neuropsychiatric Association and the Society for Behavioral and Cognitive Neurology developed a core curriculum for fellowship training in Behavioral Neurology and Neuropsychiatry, grounded in the recognition that behavioral neurology and neuropsychiatry share fundamental conceptual and clinical foundations [13].

In clinical practice, such neurologists may devote a substantial proportion of their work to cognitive disorders and aging populations. In contexts where competence is questioned, focused training, supervised exposure, and longitudinal practice are often more informative than professional labels alone.

**Clinical neuropsychology training**

Clinical neuropsychology is a recognized specialty within psychology with a distinct training model emphasizing psychometrics, standardized testing, normative interpretation, and performance validity methodology. Postdoctoral residency training is designed to produce advanced competence in these methods, particularly for comprehensive and forensic evaluations.

The APA Guidelines for Psychological Practice with Older Adults state that the comprehensive evaluation of cognitive impairment in older adults requires specialized neuropsychological training and competence, and that such evaluations should be performed by psychologists who possess relevant training, experience, and demonstrated competencies in neuropsychological assessment [8]. Neuropsychologists draw upon their understanding of age-related brain changes and the wide range of conditions that affect the brain when conducting comprehensive evaluations [8]. These statements appropriately emphasize the importance of specialized training in psychometric methods, standardized test administration, and validity assessment. However, they are framed within the context of professional guidelines specific to clinical neuropsychology and should not be interpreted as implying exclusive ownership of cognitive or neuropsychological assessment across all clinical contexts [8].

In practice, competence in cognitive and neuropsychological evaluation is not defined solely by professional discipline but by specialty training in neuroscience (i.e., neurology, psychiatry), experience, and the specific clinical tasks being performed. Physicians, particularly neurologists with focused training in cognitive disorders, may develop sufficient expertise to administer and interpret selected neuropsychological instruments within the scope of their clinical practice, particularly when the primary goal is diagnostic integration rather than comprehensive psychometric characterization. Accordingly, the appropriate standard is not professional title, but demonstrable competence aligned with the clinical question and the methods employed. These strengths complement physician training rather than replicate it. Neuropsychologists offer detailed characterization of cognitive and emotional functioning that is often not obtainable through other diagnostic modalities [7,8].

The interpretive contribution of clinical neuropsychology should therefore not be reduced to test administration or score production. Neuropsychological interpretation includes selection of instruments, estimation of premorbid ability, evaluation of reliability and validity, analysis of error patterns and domain profiles, consideration of cultural and demographic norms, assessment of functional implications, and formulation of cognitive phenotypes that may meaningfully guide differential diagnosis and management [7,8].

**The Neurological Examination and Medical Diagnosis**

**Integration of cognitive and non-cognitive findings**

The neurological examination provides essential information that cannot be obtained through cognitive testing alone. The JAMA dementia review emphasizes that the etiology of dementia is determined based on medical history, neurologic examination (including cognitive impairment, focal signs, Parkinsonism, cerebellar signs), pertinent systemic signs, neuropsychological testing, laboratory testing, biochemical markers, and brain imaging [11].

Many etiologic diagnoses in cognitive neurology depend on neurological and systemic features that cannot be inferred from cognitive testing alone. Dementia with Lewy bodies is clinically defined by prominent attentional, executive, or visuospatial deficits occurring alongside recurrent visual hallucinations, cognitive fluctuations, motor Parkinsonism, and rapid eye movement (REM) sleep behavior disorder [14]. A JAMA Neurology study found that patients with early-onset dementia with Lewy bodies exhibited more psychotic features, cognitive fluctuations, motor changes, and apathy compared with patients who had early-onset Alzheimer’s disease, and concluded that a comprehensive motor examination is essential when evaluating early-onset dementia [15].

Creutzfeldt-Jakob disease (CJD) provides another compelling illustration. A 63-year-old patient presenting with subacute cognitive decline over several weeks, including word-finding difficulty and disorientation, may show severe impairments in verbal initiative, lexical retrieval, attention, and abstract reasoning on neuropsychological testing, with a profile suggestive of frontotemporal dysfunction [16]. Based on cognitive data alone, the findings might be attributed to rapidly progressive frontotemporal dementia, autoimmune encephalitis, or atypical Alzheimer’s disease. However, neurological examination reveals stimulus-sensitive myoclonus, cerebellar ataxia, and pyramidal signs including hyperreflexia and extensor plantar responses, a constellation characteristic of prion disease but entirely invisible to cognitive testing [17,18]. The DSM-5 specifies that individuals with CJD typically present with neurocognitive deficits alongside ataxia and abnormal movements such as myoclonus, chorea, or dystonia, and that confidence in the diagnosis is greatly increased when characteristic biomarkers are also identified [17]. A prospective study of 700 patients with prion disease confirmed that gait disturbances, myoclonus, impaired smooth pursuit, and increased limb tone are among the earliest motor abnormalities, often preceding the full development of dementia [19]. In this case, MRI reveals restricted diffusion in the caudate nucleus and cortical ribbon, EEG shows periodic sharp-wave complexes, and CSF RT-QuIC is positive (93% sensitivity for confirming prion protein misfolding), establishing a diagnosis of probable sporadic CJD [18,20]. Without the neurological examination identifying myoclonus, ataxia, and pyramidal signs, the cognitive findings alone would have been insufficient to distinguish this rapidly fatal condition from treatable causes of rapidly progressive dementia.

**Biomarker integration**

Modern dementia diagnosis increasingly requires integration of cognitive findings with biomarkers. The International Working Group was the first to propose anchoring the diagnosis of Alzheimer’s disease in patients with cognitive deficits around the presence of biomarkers, enabling more accurate and earlier disease identification [21]. The ATN framework groups biomarkers into A (amyloid), T (phosphorylated tau), and N (neurodegeneration), with Alzheimer’s disease diagnosed by the presence of amyloid-β and phosphorylated tau [22].

Current European recommendations support performing a comprehensive neuropsychological evaluation prior to cerebrospinal fluid (CSF) or positron emission tomography (PET) examination, with the decision to pursue biomarker testing guided by the clinical presentation [23]. The International Working Group framework requires the identification of specific cognitive phenotypes in conjunction with abnormal biomarkers for an Alzheimer’s disease diagnosis, and cautions against overattributing cognitive decline to Alzheimer’s disease, particularly in cases involving mild cognitive impairment [23,24]. This integration of cognitive phenotyping with biomarker data represents a fundamentally medical diagnostic process that extends beyond cognitive testing alone.

**Contributions of Neurologists and Neuropsychologists**

Neurologists contribute medical competencies that are central when the clinical question requires disease-level diagnosis, etiologic attribution, neurological examination, integration of imaging and biomarkers, and medical management of conditions such as seizures, headache syndromes, sleep disorders, autonomic dysfunction, and neurodegenerative disease. The relative involvement of neurologists, neuropsychologists, geriatricians, psychiatrists, physiatrists, speech-language pathologists, occupational therapists, and other clinicians varies across healthcare systems, institutional resources, referral pathways, and organizational models. These medical competencies are not substitutes for psychometric expertise, and they do not imply that neurologists should perform all forms of cognitive evaluation. Rather, they identify the elements of assessment that require medical training and neurological integration. The Lancet Neurology review on young-onset dementia notes that while bedside cognitive examination can provide useful information, formal neuropsychological assessment is needed to characterize the patient’s cognitive syndrome in greater detail [25].

Neuropsychologists contribute interpretive and diagnostic strengths that are difficult to replicate in standard medical encounters, including psychometric breadth, performance validity assessment, fine-grained cognitive profiling, syndrome-level characterization, detailed functional recommendations, and longitudinal measurement using consistent methodology [7,8]. These contributions are particularly valuable when measurement precision and interpretability are essential. In dementia evaluations, neuropsychological findings may identify cognitive phenotypes, distinguish patterns of impairment, support DSM-based diagnosis of neurocognitive disorders, quantify severity, and generate hypotheses that guide medical differential diagnosis (Table 2).

Performance validity testing is a specialized area in which neuropsychological expertise is essential. Performance validity tests (PVTs) are widely employed to quantify effort and detect negative response bias during neuropsychological testing [26]. However, PVT failure occurs commonly across a range of clinical conditions, even when there is no apparent incentive to underperform, requiring sophisticated interpretation that accounts for clinical context [26]. Both disciplines share competencies in recognizing cognitive syndromes, using standardized instruments within competence, identifying psychiatric comorbidity, and communicating findings meaningfully. A collaborative model avoids false dichotomies and best serves patients.

**Traumatic Brain Injury as a Model Condition**

TBI provides a useful model for clarifying roles because it is multidomain, often subtle on routine imaging, and frequently evaluated longitudinally. In older adults, TBI commonly intersects with vascular disease, medication effects, sleep disorders, sensory impairment, and emerging neurodegeneration, producing complex phenotypes.

**Diagnosis and testing in TBI**

TBI is a medical diagnosis anchored in the injury mechanism and its neurological effects. The DSM-5 specifies that the diagnosis of major or mild neurocognitive disorder due to TBI depends on performance across domain-specific cognitive assessments, interpreted in light of the individual’s prior functioning, such as neuropsychological estimates of pre-injury cognitive ability or appropriate normative data, together with an assessment of functional status [17]. Importantly, while neuroimaging and other clinical assessments, such as subtle neurological signs, may provide supportive information, they cannot independently establish the diagnosis of neurocognitive disorder due to TBI [17].

The diagnosis may be supported by subtle neurological signs, including multiple primitive reflexes such as the glabellar sign, snout response, and palmomental reflex, as well as deficits in saccades and smooth-pursuit eye movements occurring alongside frontally mediated cognitive impairments, or coordination and balance findings that require neurological examination [27,28].

**Limitations of neuropsychological testing in TBI diagnosis**

The 2025 Action Collaborative on Traumatic Brain Injury Care guideline states that neuroimaging and neuropsychological testing are neither necessary nor routinely performed to confirm or exclude a diagnosis of TBI in the post-acute stage [29]. Clinical practice guidelines generally advise against comprehensive neuropsychological assessment during the active recovery period, as cognitive recovery following TBI may take one to three months [29].

The VA/DoD guideline found no evidence supporting the routine use of comprehensive neuropsychological testing to diagnose mild TBI, guide treatment decisions, or improve outcomes in the post-acute period, and recommends against routine comprehensive neuropsychological testing in this population within the first 30 days [30].

A recent review concluded that traditional neuropsychological methods were not originally developed to detect subtle neurocognitive or neurobehavioral changes as a standalone procedure, nor were they specifically designed to assess the effects of mild TBI [31]. This underscores that neuropsychological testing characterizes cognitive sequelae but is not determinative of whether injury occurred.

**When neuropsychological assessment adds essential value**

Collaboration with a neuropsychologist in neurological disorders affecting cognition is essential when developmental or educational factors complicate interpretation, including intellectual disability, borderline intellectual functioning, illiteracy, very low educational attainment, or when visual or auditory impairments complicate standard assessment methods. In such cases, specialized psychometric techniques and normative interpretation are required to avoid misclassification.

Neuropsychological expertise is critical when sensory, motor, or oculomotor limitations constrain standard testing. Visual impairment, diplopia, impaired eye movements, tremor, or vestibular symptoms may invalidate conventional tasks, necessitating alternative modalities such as verbally mediated or modified-response testing [8,32].

Performance validity assessment is another domain where neuropsychological training is essential, particularly in medicolegal contexts. The APA Guidelines on Assessment of and Intervention with Persons with Disabilities note that the purpose of validity assessment is to determine whether the individual has exerted sufficient effort to perform well, thereby preventing possible overrepresentation of need, or, conversely, has exaggerated responses, which could result in underrepresentation of need [32]. However, behavioral observations and clinical judgment alone are not sufficient to make validity determinations [32].

Neuropsychological assessment also adds value when fine-grained cognitive phenotyping is needed to inform differential diagnosis in aging populations or when formal functional determinations are required. The TBI guideline states that referral for a formal neuropsychological assessment, or cognitive assessment by another provider if neuropsychology is not accessible, may be offered to inform planning for return to sport, school, or work, or to guide treatment in patients with persisting cognitive symptoms beyond one month [29].

**Neurologist-led evaluation of cognitive function**

Neurologist-led cognitive assessment may be sufficient when the primary question is diagnostic or management-focused, when cognitive complaints occur in the absence of the above-mentioned factors, or when non-cognitive neurological impairments dominate disability. In these scenarios, medical integration rather than psychometric density is central.

The AAN Behavioral Neurology Section Workgroup identified single-domain cognitive tests suitable for office-based neurological practice, emphasizing that standardized cognitive tests, particularly those with normative data based on the individual’s age and educational level, can enhance the rigor and clinical utility of cognitive assessment [5].

**Normal cognitive or neuropsychological testing does not exclude TBI**

Normal cognitive or neuropsychological test performance does not exclude TBI [17,29,31]. Many disabling post-TBI sequelae are non-cognitive, cognitive inefficiencies may be state-dependent, and high premorbid ability may mask deficits. Tests provide a partial window into brain function and must be interpreted within a broader evaluation.

The DSM-5 notes that performance on commonly used general cognitive screening measures, particularly when interpreted using large-scale, population-based normative data, may usefully identify individuals who require further neurodiagnostic assessment, but emphasizes that diagnosis requires integration with functional status and clinical context [17].

**The Intersection of Aging, Neurodegeneration, and TBI**

Aging expands interpretive complexity because cognitive symptoms after TBI frequently intersect with neurodegenerative disease, vascular pathology, medication effects, sleep disorders, and mood symptoms. Neuropsychological assessment can characterize phenotype and track change, but etiologic attribution requires a neurological evaluation and medical differential diagnosis, particularly when non-cognitive features suggest early frontotemporal dementia, Lewy body disease, Parkinsonian syndromes, or prionosis.

Similarly, the JAMA Neurology study on cognitive phenotyping and Alzheimer’s blood biomarkers found that cognitive phenotyping is essential for accurately interpreting blood p-tau217 results in clinical practice [33]. Different cognitive phenotypes exhibit varying amyloid prevalence and predictive values for biomarker testing, underscoring the importance of detailed cognitive characterization within a medical diagnostic framework.

**Practical Implications and Collaborative Model**

An appropriate clinical and forensic approach must be individualized, method-matched, scope-matched, and responsive to local healthcare-system resources and legal or organizational constraints. Neuropsychological assessment is used when psychometric depth or validity analysis is required; medical-neurological evaluation remains central when disease-level diagnosis, etiologic attribution, and neurological examination determine the clinical question. In many settings, these processes occur concurrently rather than sequentially within multidisciplinary memory clinics, rehabilitation programs, or collaborative referral networks.

**Scope of medicolegal implications**

The medicolegal implications addressed in this review refer primarily to the interpretation of cognitive findings, professional competence, and the evidentiary limits of test performance when making neurological diagnoses. The paper does not attempt to provide a comprehensive legal framework for capacity determinations, disability certification, fitness-for-duty decisions in safety-sensitive occupations, professional licensure reporting, driving restrictions, or jurisdiction-specific dementia reporting requirements. Those determinations depend on the legal question being asked, the patient’s role and occupational demands, state or national law, and often specialized functional, occupational, or neuropsychological assessment. Empirical evidence most directly relevant to this review concerns validity assessment, limitations of relying on test scores alone, and the need to interpret cognitive performance in clinical context. In such settings, the neurologist provides medical diagnosis, assessment of neurological contributors, and explanation of functional neurological limitations, while neuropsychological assessment may provide additional psychometric and performance-validity data when the legal or occupational question requires that level of measurement precision [8,32].

The Journal of Neurology, Neurosurgery, and Psychiatry review notes that neurologists often find it difficult to interpret neuropsychological test results, despite cognitive assessments being an integral component of the diagnostic process in dementia syndromes [34]. This observation supports a collaborative model where neuropsychologists provide detailed cognitive characterization, and neurologists integrate findings within the broader diagnostic framework.

The tiered model proposed here is not intended as a claim of de novo novelty or as a replacement for established AAN, APA, NAN, VA/DoD, dementia, or neuropsychological practice guidelines. Its purpose is to operationalize their complementary implications into a practical, method-matched workflow for clinicians who must decide what level of cognitive or neuropsychological assessment is appropriate for a given clinical question. The model should be applied flexibly because referral patterns, professional roles, and available resources differ across healthcare systems and organizational settings. Based on the foregoing discussion, we propose a tiered, collaborative model for aging populations and patients with TBI:

**Tier 1:** Neurologist-led cognitive assessment using validated screening and single-domain tests (NBSE) for diagnostic and management questions, with integration of neurological examination, imaging, and biomarkers [5,11].

**Tier 2:** Referral for comprehensive neuropsychological assessment when developmental/educational factors complicate interpretation, sensory/motor limitations constrain testing, performance validity is a concern, fine-grained cognitive phenotyping is needed, or formal functional determinations are required [8,29,32].

**Figure 1:**
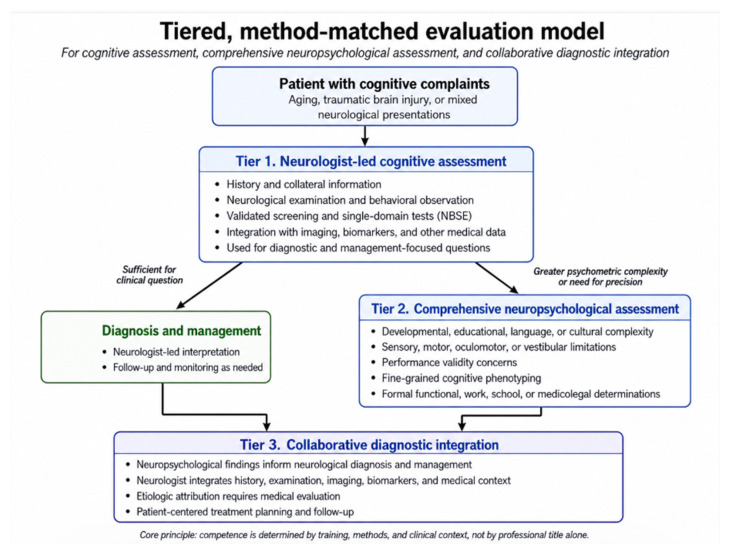
Tiered, method-matched evaluation model for cognitive assessment, comprehensive neuropsychological assessment, and collaborative diagnostic integration.

**Tier 3:** Collaborative integration where neuropsychological findings inform neurological diagnosis and management, with recognition that etiologic attribution requires medical evaluation [11,25] (Figure 1).

## 2. Limitations

This paper does not substitute for medical boards’ regulatory rules or jurisdiction-specific legal guidance on testing outside the fields of neurology or neuropsychology. Scope-of-practice boundaries vary by law and regulation, and individual competence varies by specialty, training, experience, local credentialing, and institutional policy. Because direct comparative studies of professional groups are limited, statements about professional roles should be understood as a synthesis of guidelines, training standards, regulatory frameworks, and clinical reasoning rather than as conclusions from head-to-head outcome trials. The framework presented describes clinical and diagnostic expectations rather than universal hard rules. In addition, this article is not a systematic review or meta-analysis of the diagnostic accuracy of cognitive tests for dementia, nor is it intended as a comprehensive review of reversible or modifiable contributors to cognitive symptoms such as medication effects, sleep deprivation, obstructive sleep apnea, sensory impairment, language barriers, psychiatric disease, pain, or fatigue. These factors are clinically important and should be evaluated when relevant, but detailed algorithms for their assessment and treatment would require separate focused reviews. Jurisdiction-specific dementia reporting requirements, driving restrictions, licensure reporting, occupational fitness-for-duty standards, and capacity determinations also require specialized legal and functional frameworks beyond the scope of this review.

Digital and artificial-intelligence-based cognitive assessment tools are also an important emerging area. A recent systematic review and meta-analysis of digital tools for mild cognitive impairment reported generally promising pooled diagnostic performance but substantial heterogeneity, methodological variability, applicability concerns, and demographic influences. These methods may eventually augment screening, monitoring, or triage, but they do not change the central argument of this review: cognitive data, whether obtained through paper-based testing, computerized testing, or AI-assisted tools, require validation, appropriate norms, validity assessment when indicated, and clinical-medical integration before etiologic conclusions are drawn [35,36].

Although this review did not perform a quantitative meta-analysis, existing meta-analytic evidence on memory measures shows high diagnostic accuracy for Alzheimer’s dementia and more limited diagnostic accuracy for mild cognitive impairment, illustrating that the relevant empirical literature primarily evaluates instruments and cognitive constructs rather than professional discipline [37].

Similarly, cross-cultural, linguistic, and socioeconomic factors are discussed only to the extent necessary to support the competence-based framework. Dedicated reviews and position statements provide more detailed treatment of culturally and educationally diverse cognitive assessment, including the need for culturally appropriate instruments, interpreter-mediated assessment, and norms that account for language, education, and literacy [3,4].

## 3. Conclusions

Neurologists are trained to evaluate cognitive symptoms within a medical and neurological framework and to provide diagnoses involving cognition when the clinical presentation requires disease-level etiologic attribution. The AAN Behavioral Neurology Section Workgroup’s development of the NBSE framework was explicitly aimed at improving the quality of clinical cognitive assessment by neurologists through the use of standardized tests with normative data [5]. Clinical neuropsychologists contribute essential expertise for more granular psychometric assessment, culturally and demographically informed normative interpretation, functional characterization, cognitive phenotyping, and performance validity analysis, particularly in complex clinical and medicolegal settings [8,26,32]. These complementary contributions should be understood as differences in training focus and clinical method, not as a hierarchy of professional value.

Both neurologists and neuropsychologists may be qualified to perform cognitive and, in appropriate circumstances, neuropsychological testing, provided they possess the specific training, experience, methodological competence, and local authorization required for the procedures being performed and interpreted. This statement is not intended to equate training pathways, but to emphasize that competence is method-specific and clinically contextual. For neurologists, this requires expertise or experience when complex psychometric batteries, validity methods, or highly specialized normative interpretation are involved.

Neuropsychologists, while highly specialized in cognitive and behavioral assessment, are qualified to diagnose DSM-based neurocognitive disorders based on standardized cognitive and behavioral criteria [2,8]. When the clinical question is attribution to a specific neurological etiology (e.g., Alzheimer’s disease, Lewy body disease, frontotemporal degeneration, Creutzfeldt-Jakob disease, or traumatic brain injury), the cognitive diagnosis must be integrated with medical evaluation, including neurological examination, assessment of non-cognitive clinical features, and, when appropriate, imaging or biomarker data [11,14,17,18,24]. These determinations depend on the integration of clinical domains that extend beyond cognitive testing alone.

## Figures and Tables

**Table 1:** Practical distinctions among cognitive screening, integrated cognitive assessment, and comprehensive neuropsychological assessment.

Feature	Cognitive Screening	Integrated Cognitive Assessment	Comprehensive Neuropsychological Assessment
Primary purpose	Detect possible impairment and determine whether more evaluation is needed.	Answer a clinical diagnostic or management question by integrating cognition with medical and neurological context.	Provide detailed psychometric characterization of cognitive, behavioral, emotional, and functional status.
Typical methods	Brief tools such as MMSE, MoCA, Mini-Cog, or domain-limited screens.	History, collateral information, neurological examination, functional review, targeted standardized tests, and relevant medical data.	Standardized multi-domain batteries, norm-referenced interpretation, validity measures, error-pattern analysis, and functional recommendations.
Interpretive emphasis	Sensitivity to global impairment; limited etiologic specificity.	Clinical localization, differential diagnosis, comorbidity, functional consequences, and treatment planning.	Cognitive profile, severity, pattern of strengths and weaknesses, validity, and functional implications.
Best-suited questions	Should cognitive concerns be evaluated further?	What medical or neurological processes best explain the patient’s presentation?	What is the detailed cognitive phenotype and how valid, severe, and functionally relevant are the findings?
Limitations	Ceiling/floor effects; limited sensitivity for subtle MCI or high premorbid ability; vulnerable to education/language effects.	May be insufficient when complex norms, validity assessment, or fine-grained psychometrics are required.	Does not by itself establish medical etiology; requires integration with neurological, systemic, imaging, and biomarker data when etiology is at issue.

**Table 2:** Complementary contributions of neurologists and clinical neuropsychologists in cognitive evaluation.

Clinical Domain	Neurologist Contribution	Clinical Neuropsychologist Contribution	Integrated Value
Medical diagnosis and etiology	Links symptoms to neurological examination, systemic illness, imaging, biomarkers, medications, sleep, and disease mechanisms.	Provides cognitive phenotype and behavioral data that may support or challenge diagnostic hypotheses.	Reduces overreliance on test scores or clinical impression alone.
Psychometrics and norms	Uses validated screening and targeted instruments within competence and clinical context.	Selects and interprets comprehensive batteries with demographic, cultural, and premorbid adjustments.	Improves precision and reduces misclassification.
Performance validity	Recognizes contextual factors and when validity assessment is needed.	Applies and interprets embedded and stand-alone validity measures, especially in forensic settings.	Improves interpretability and evidentiary reliability.
Cross-cultural and language issues	Identifies when language, literacy, education, sensory impairment, or socioeconomic context may affect results.	Adapts assessment strategy, norms, and interpretation when specialized methods are required.	Supports fairer interpretation in diverse populations.
Functional and occupational questions	Defines neurological impairments, medical restrictions, prognosis, and treatment implications.	Quantifies cognitive strengths, weaknesses, and functional consequences when formal measurement is required.	Supports role-specific recommendations without confusing diagnosis with legal capacity.
Management and follow-up	Treats medical contributors and integrates findings with longitudinal disease course.	Tracks cognitive change and provides rehabilitation, compensatory, and caregiver recommendations.	Promotes patient-centered diagnosis and care planning.

## Data Availability

No new data were generated or analyzed in this review.

## Supplementary Materials

The following supporting information can be downloaded at: https://www.mdpi.com/article/10.3390/jcm15103822/s1, File S1: Regulatory and reimbursement framework for neuropsychological testing by physicians [38,39,40,41,42,43,44,45,46].
