# Supplementary material for: Evaluating Cognition Across Aging and Traumatic Brain Injury: Integrating Neurological and Neuropsychological Approaches
Source: J Clin Med. 2026 May 15;15(10):3822. doi: 10.3390/jcm15103822 (PMC13207243; doi:10.3390/jcm15103822)
Supplement: Supplementary file 1 [file jcm-15-03822-s001.zip › jcm-4301349-supplementary.pdf]

Supplementary File S1: Regulatory and Reimbursement Laws and Regulations Supporting Neuropsychological Testing by Physicians

S.1 Introduction

The main body of this paper establishes a competence-based framework for cognitive and neuropsychological assessment. This appendix provides supporting evidence from regulatory, reimbursement, and professional practice domains demonstrating that the administration and interpretation of neuropsychological tests by physicians, including neurologists, is recognized and supported by multiple authoritative bodies. These include the Centers for Medicare and Medicaid Services (CMS), the American Medical Association (AMA) Current Procedural Terminology (CPT) system, third-party health insurance payers, state medical practice acts, and professional organizations in both neurology and neuropsychology.

S.2 CMS/Medicare Reimbursement Framework

S.2.1 CPT Code Structure and Physician Eligibility

The AMA CPT coding system, which serves as the foundation for Medicare and most third-party reimbursement, explicitly recognizes physicians as eligible providers for neuropsychological testing services. The 2019 CPT code restructuring created a tiered system that distinguishes between evaluation/interpretation and test administration, with specific codes designated for physicians and qualified healthcare professionals (QHPs):

Evaluation and Interpretation Codes (Physician/QHP):

- CPT 96116: Neurobehavioral status examination by physician or other qualified healthcare professional, first hour (face-to-face with patient), with interpretation and report [S1].

- CPT 96121: Neurobehavioral status examination, each additional hour (add-on to 96116) [S1].

- CPT 96132: Neuropsychological testing evaluation services by physician or other qualified healthcare professional, first hour (face-to-face with patient), including integration of patient data, interpretation of standardized test results and clinical data, clinical decision making, treatment planning, and report, and interactive feedback to the patient, family

member(s), or caregiver(s) [S1].

- CPT 96133: Neuropsychological testing evaluation services, each additional hour (add-on to 96132) [S1].

Test Administration Codes (Physician/QHP):

- CPT 96136: Psychological or neuropsychological test administration and scoring by physician or other qualified healthcare professional, two or more tests, any method, first 30 minutes [S1].

- CPT 96137: Each additional 30 minutes (add-on to 96136) [S1].

Test Administration Codes (Technician):

- CPT 96138: Psychological or neuropsychological test administration and scoring by technician, two or more tests, any method, first 30 minutes [S1].

- CPT 96139: Each additional 30 minutes (add-on to 96138) [S1].

This coding structure is significant for several reasons. First, the CPT system explicitly uses the term "physician or other qualified healthcare professional" for codes 96116, 96121, 96132, 96133, 96136, and 96137, rather than restricting these codes to psychologists or neuropsychologists. Under AMA CPT definitions, a "physician or other qualified healthcare professional" is an individual who is qualified by education, training, licensure/regulation, and facility privileging to perform a specific service [A1]. This definition encompasses neurologists and other physicians who possess the requisite training and competence.

Second, the distinction between physician/QHP administration codes (96136–96137) and technician administration codes (96138–96139) confirms that the CPT system contemplates direct test administration by physicians, not merely interpretation of tests administered by others.

Third, the neurobehavioral status examination codes (96116, 96121) are categorized within the Medicine , Neurology section of the CPT manual, reflecting their origin in and relevance to neurological practice [S1].

S.2.2 Medicare Physician Fee Schedule

The CMS Medicare Physician Fee Schedule (MPFS) assigns work relative value units (wRVUs) to neuropsychological testing codes and reimburses these services when billed by eligible providers. A study examining wRVUs for neuropsychological evaluation CPT codes (96116, 96132, 96133, 96136, and 96137) confirmed that these codes are included in the CMS fee schedule with assigned wRVUs and reimbursement rates based on the CMS conversion factor [S2]. The MPFS does not restrict reimbursement for these codes to psychologists; physicians who meet the CPT definition of a qualified healthcare professional are eligible to bill these services.

CMS has historically recognized that neuropsychological and psychological testing codes may be billed by multiple provider types. Confusion regarding billing practices has been documented in the professional literature, with one commentary noting that much of this confusion has stemmed from discrepancies in interpretation of testing codes between the AMA and CMS, and that clinicians must ultimately use codes according to the requirements of the specific payor [S3]. This observation underscores that the regulatory framework permits physician billing but that individual payor policies may impose additional requirements.

A.2.3 Medicare Cognitive Assessment and Care Planning

In 2017, Medicare introduced specific reimbursement codes for cognitive assessment and care planning (CACP), further establishing physicians' role in structured cognitive evaluation. These codes require cognition-focused evaluation, identification of caregivers and caregiver needs, and development of an advanced care plan [S4]. The CACP codes are available to physicians across specialties, including neurologists, and represent a federal recognition that cognitive assessment is within the scope of practice for physicians.

Additionally, the Medicare Annual Wellness Visit, established under the Patient Protection and Affordable Care Act, requires detection of cognitive impairment as a mandatory component [S5][S6]. This federal mandate applies to all eligible Medicare providers, including physicians, and does not restrict cognitive assessment to psychologists or neuropsychologists.

S.3 Third-Party Insurance Payer Practices

S.3.1 Private Insurer Reimbursement Models

Private health insurance payers have broadly adopted the AMA CPT coding framework for neuropsychological testing services. Because the CPT codes designate "physician or other qualified healthcare professional" as eligible providers, private insurers who follow the CPT framework implicitly recognize physician eligibility for these services. Many private insurers and state Medicaid providers have authorized neuropsychological CPT codes for reimbursement across provider types [S7].

The reimbursement landscape for neuropsychological services has been characterized by variability across payors. A national survey of clinical neuropsychologists found that numerous CPT codes are used to bill the same clinical service, and that awareness of Medicare practice and billing expectations is variable among practitioners [S8]. This variability extends to private insurers, where managed care companies may impose different coverage policies, but the underlying CPT code structure, which permits physician billing, remains consistent.

S.3.2 State Medicaid Programs

State Medicaid programs generally follow CMS guidelines for provider eligibility and CPT code usage, though individual states may impose additional requirements. Many state Medicaid carriers have coverage policies that specify they follow CMS policy regarding eligible providers for neuropsychological testing services [S7]. This alignment with federal policy further supports the recognition of physicians as eligible providers for neuropsychological testing.

S.4 Professional and Regulatory Framework

S.4.1 State Medical Practice Acts

State medical practice acts broadly define the scope of physician practice to include the diagnosis and treatment of diseases and conditions affecting the nervous system, which encompasses cognitive evaluation. Physicians licensed under state medical practice acts are generally authorized to perform any medical service for which they possess adequate training and competence, without the need for separate licensure in psychology or neuropsychology. The practice of medicine, as defined by state medical boards, includes evaluating brain function through standardized testing when performed within the physician's scope of competence.

This legal framework is distinct from psychology licensure laws, which govern the practice

of psychology by non-physicians. Importantly, state medical practice acts do not typically exclude cognitive or neuropsychological testing from the scope of physician practice; rather, they establish that physicians may perform any service within their competence as defined by their medical training and licensure.

S.4.2 ABPN Board Certification

As discussed in the main text, the American Board of Psychiatry and Neurology (ABPN) includes neuropsychological and cognitive testing among the diagnostic procedures relevant to neurology board certification.[1] This inclusion reflects the expectation that board-certified neurologists possess foundational knowledge of neuropsychological testing methods and their application in clinical practice.

S.4.3 AAN Practice Framework

The American Academy of Neurology (AAN) Behavioral Neurology Section Workgroup explicitly developed the Neurobehavioral Status Exam (NBSE) framework to improve the quality of clinical cognitive assessment performed by neurologists.[2] The workgroup identified standardized cognitive tests with normative data suitable for office-based neurological practice, establishing a professional standard for neurologist-administered cognitive testing that extends beyond brief screening.[2]

S.4.4 AACN Practice Guidelines

The American Academy of Clinical Neuropsychology (AACN) Practice Guidelines for Neuropsychological Assessment and Consultation, while primarily directed at clinical neuropsychologists, acknowledge that applicable federal and state laws supersede professional guidelines [S9]. The guidelines define the clinical neuropsychologist as a psychologist who engages in the practice of clinical neuropsychology, but do not assert that neuropsychological testing is exclusively restricted to psychologists [A9]. The guidelines further note that other organizations, disciplines, professionals, entities, and individuals are encouraged to consider these guidelines as principles for the provision of neuropsychological services [S9].

S.4.5 Research Practice Standards

The USPSTF systematic review on screening for cognitive impairment in older adults documented that in research settings, the reference standard for diagnosing dementia or

MCI , which typically consisted of a neuropsychological battery of tests , was administered by research staff, neurologists, psychiatrists, psychologists, psychometricians, other physicians, and nurses, with diagnosis usually made by consensus [S10]. This finding confirms that neuropsychological test administration by neurologists and other physicians is an established and accepted practice in clinical research, further supporting its legitimacy in clinical care.

S.5 Summary of Regulatory Support

The regulatory and reimbursement evidence converges on several key conclusions that align with the competence-based framework proposed in this paper:

1. The AMA CPT coding system explicitly designates physicians as eligible providers for neuropsychological testing evaluation, interpretation, and administration codes, using the term "physician or other qualified healthcare professional" rather than restricting these services to psychologists [S1].

2. The CMS Medicare Physician Fee Schedule reimburses neuropsychological testing codes when billed by eligible physicians, and Medicare has established specific cognitive assessment codes available to physicians across specialties [S2][S4].

3. Third-party insurance payers have broadly adopted the CPT framework, which permits physician billing for neuropsychological services, though individual payor policies may vary [S7][S8].

4. State medical practice acts generally authorize physicians to perform cognitive and neuropsychological testing within their scope of competence, without requiring separate psychology licensure [S9].

5. Professional organizations in both neurology and neuropsychology have developed frameworks that support physician-administered cognitive testing, with the AAN explicitly developing standardized approaches for neurologist-led cognitive assessment.[2]

6. Research practice standards confirm that neuropsychological test administration by neurologists and other physicians is an established practice in clinical research [S10].

These regulatory and reimbursement structures are consistent with the competence-based model advocated in this paper: the appropriate standard for performing

neuropsychological testing is not professional title but demonstrable competence aligned with the clinical question and the methods employed.

Appendix References

S1. American Medical Association. Current Procedural Terminology (CPT) 2019 Professional Edition. American Medical Association; 2018.

S2. Steel SA, Rolin SN, Davis JJ. Relatively Undervalued: Comparing the Work Relative Value Units of Neuropsychological Evaluation to Other Services. The Clinical Neuropsychologist. 2024;38(4):907-921. doi:10.1080/13854046.2023.2272788.

S3. Rosenstein LD. Commentary on the Use of 96119 in Billing for Neuropsychological Services. The Clinical Neuropsychologist. 2017;31(8):1273-1282. doi:10.1080/13854046.2017.1329459.

S4. Li J, Andy C, Mitchell S. Use of Medicare's New Reimbursement Codes for Cognitive Assessment and Care Planning, 2017-2018. JAMA Network Open. 2021;4(9):e2125725. doi:10.1001/jamanetworkopen.2021.25725.

S5. Jacobson M, Thunell J, Zissimopoulos J. Cognitive Assessment at Medicare's Annual Wellness Visit in Fee-for-Service and Medicare Advantage Plans. Health Affairs. 2020;39(11):1935-1942. doi:10.1377/hlthaff.2019.01795.

S6. Patient Protection and Affordable Care Act, Pub. L. No. 111-148, 124 Stat. 119 (2010).

S7. Bilder RM, Postal KS, Barisa M, et al. Inter Organizational Practice Committee Recommendations/Guidance for Teleneuropsychology in Response to the COVID-19 Pandemic. Archives of Clinical Neuropsychology. 2020;35(6):647-659. doi:10.1093/arclin/acaa046.

S8. Sweet JJ, Peck EA, Abramowitz C, Etzweiler S. National Academy of Neuropsychology/Division 40 of the American Psychological Association Practice Survey of Clinical Neuropsychology in the United States. Part II: Reimbursement Experiences, Practice Economics, Billing Practices, and Incomes. Archives of Clinical Neuropsychology. 2003;18(6):557-582.

S9. American Academy of Clinical Neuropsychology. AACN Practice Guidelines for

Neuropsychological Assessment and Consultation. The Clinical Neuropsychologist. 2007;21(2):209-231. doi:10.1080/13825580601025932.

S10. Patnode CD, Perdue LA, Rossom RC, et al. Screening for Cognitive Impairment in Older Adults: Updated Evidence Report and Systematic Review for the US Preventive Services Task Force. JAMA. 2020;323(8):764-785. doi:10.1001/jama.2019.22258.

Additional General References

1. Improving Clinical Cognitive Testing: Report of the AAN Behavioral Neurology Section Workgroup. Daffner KR, Gale SA, Barrett AM, et al. Neurology. 2015;85(10):910-8. doi:10.1212/WNL.0000000000001763.

2. Cognitive Screening Tests Versus Comprehensive Neuropsychological Test Batteries: A National Academy of Neuropsychology Education Paper†. Roebuck-Spencer TM, Glen T, Puente AE, et al. Archives of Clinical Neuropsychology : The Official Journal of the National Academy of Neuropsychologists. 2017;32(4):491-498. doi:10.1093/arclin/acx021.
